# Advances in the Study of Antitumour Immunotherapy for Newcastle Disease Virus

**DOI:** 10.7150/ijms.59185

**Published:** 2021-03-30

**Authors:** Qiuxing Meng, Jian He, Liping Zhong, Yongxiang Zhao

**Affiliations:** 1 The First Affiliated Hospital of Guangxi Medical University, Nanning, Guangxi, China.; 2 National Center for International Research of Bio-targeting Theranostics, Guangxi Key Laboratory of Bio-targeting Theranostics, Collaborative Innovation Center for Targeting Tumor Diagnosis and Therapy, Guangxi Talent Highland of Bio-targeting Theranostics, Guangxi Medical University, Nanning, Guangxi, China.

**Keywords:** Newcastle disease virus, Immunotherapy, Tumour, Immune checkpoint inhibitors, Genetic engineering technology, Recombinant NDV

## Abstract

This article reviews the preclinical research, clinical application and development of Newcastle disease virus (NDV) in the field of cancer therapy. Based on the distinctive antitumour properties of NDV and its positive interaction with the patient's immune system, this biologic could be considered a major breakthrough in cancer treatment. On one hand, NDV infection creates an inflammatory environment in the tumour microenvironment, which can directly activate NK cells, monocytes, macrophages and dendritic cells and promote the recruitment of immune cells. On the other hand, NDV can induce the upregulation of immune checkpoint molecules, which may break immune tolerance and immune checkpoint blockade resistance. In fact, clinical data have shown that NDV combined with immune checkpoint blockade can effectively enhance the antitumour response, leading to the regression of local tumours and distant tumours when injected, and this effect is further enhanced by targeted manipulation and modification of the NDV genome. At present, recombinant NDV and recombinant NDV combined with immune checkpoint blockers have entered different stages of clinical trials. Based on these studies, further research on NDV is warranted.

## Introduction

Cancer is a disease that is difficult for human beings to conquer. Countless researchers devote themselves to developing new treatment regimens to improve the survival rate of cancer patients. At present, the three major treatment methods are surgical resection, radiotherapy and chemotherapy, but these treatments have high side effects and lack targeted therapy. Therefore, new cancer treatment methods have yet to be explored.

As early as the beginning of the nineteenth century, it was discovered that severe viral infections can lead to temporary remission in human leukemia. It was understood at the time that the virus directly or indirectly infected tumour cells, leading to the relief of tumour symptoms. Subsequently, a number of clinical trials began to search for viruses that could selectively lyse tumor cells with limited pathogenicity in humans. In 1964 1, the first case report of acute leukemia treated with Newcastle disease virus (NDV) was published. The patient experienced transient hematologic and clinical remission followed administration of NDV. In 1965 2, for the first time, Cassel and Garret from Atlanta used NDV (73T virus strain) as an antitumour drug when adenovirus and NDV were injected directly into uterine cancer, causing the tumour to partially necrotize and shade, but it regrew quickly. The reason for this short-term oncolytic effect may be that considering the virulence of wild type viruses, virus titers used clinically are usually low. As a result, the virus is cleared by the patient's immune system before it can be effectively lysed, causing the residual tumour to grow back quickly. Although these early NDVs usually produce only short-term lytic effects and are accompanied by toxic side effects from wild type viral infections, many studies have shown the possibility of NDV as a therapeutic agent in cancer treatment, both in mouse model studies and in human clinical trials 3-5.

The total length of the NDV genome is 15186 bp, which contains 6 genes encoding at least 8 proteins in the order of NP-P/V/W-M-F-HN-L, including six structural proteins - nucleoprotein (NP), phosphoprotein (P), matrix protein (M), fusion protein (F), haemagglutinin neuraminidase (HN) and polymerase protein (L) - and two additional proteins, V and W. Among the structural proteins, NP, P, and L bind to viral RNA to form ribonucleoprotein complex (RNP), which are responsible for viral replication 6. HN and F exist in the form of oligomers, which, together with the host lipid bilayer, constitute the outer membrane of the virus and participate in the process of virus entry into the cell 7, 8. F is usually present in the form of the inactive polypeptide F0, which is cleaved into F1 and F2 to acquire infectivity. The V protein, as a non-structural protein, is thought to be an interferon antagonist and is also associated with the virulence of NDV viruses 9. Recent studies have shown that the V protein can mediate the promotion of NDV replication by participating in the ERK signalling pathway 10, while M is related to virus assembly and germination 11, 12.

NDV is a paramyxovirus that can infect birds to cause Newcastle disease. According to the pathogenicity in one-day-old chickens, NDV can be divided into three types: velogenic (highly virulent), mesogenic (moderate virulent) and lentogenic (low virulent). In general, the virulence of NDV is related to the cleavage site of the F protein 13. The F protein is a class I transmembrane protein synthesized by precursor protein F0, which is cleaved by cellular proteases into disulphide-linked subunits F1 and F2 that become proteins necessary for viral infectivity in offspring. The mesogenic and velogenic strains of NDV have a multi-base amino acid (AA) motif, 112(K/R)-R-(Q/K)-(R/K)-R116, at the carboxy-terminus of F2 and a phenylalanine at the amino terminus of the F1 subunit (AA117), which can be recognized and cleaved by forin-like proteases (RXK/RR), leading to systemic infection reactions, such as MTH-68/H and PV701. In contrast, because the F protein of the weak strain is characterized by a leucine at position 117 and a single-group AA motif at the carboxy-terminus of F2112(G/E)-(K/R)-Q-(G/E)R116, the virus can only be treated by trypsin-like enzymes restricted to the respiratory and intestinal tracts, resulting in mild respiratory and gastrointestinal disease. At present, the commonly used strains in preclinical studies are medium strains MTH-68/H, PV701, AF2240 and 73T, as well as weak strains HUJ, Lasota and Ulster.

## NDV as a cancer vaccine

Among oncolytic viruses, NDV belongs to the group of attenuated strains of wild-type oncolytic viruses. Compared with viruses from other families, the use of NDV has the following advantages. First, its replication process occurs in the cytoplasm, from RNA to RNA, and there is no possibility of a DNA phase or cellular genome integration. This cytoplasmic replication makes the virus independent of the host cell DNA replication machinery, and cellular genome integration is impossible; thus, patients with cancer can tolerate even relatively high doses of NDV. Currently, three main routes of NDV administration in the human body have been developed: intratumor injection, intravenous injection and nasal inhalation. According to previous reports 14-16, the intratumor route uses less than 4×10^12^ infection units, and the intravenous route uses less than 3×10^9^ infection units, which are well tolerated. Based on these clinical data, NDV infection will generate fewer side effects. Patients usually present with mild influenza-like symptoms such as fatigue, headache, weakness and fever. Erythema, swelling, sclerosis and pruritus have been observed at the site of inoculation in some patients. Occasionally, patients experience transient thrombocytopenia or neutropenia and diffuse vascular leakage. However, these side effects are temporary and disappear within one to two days of vaccination.

**Figure 1:**
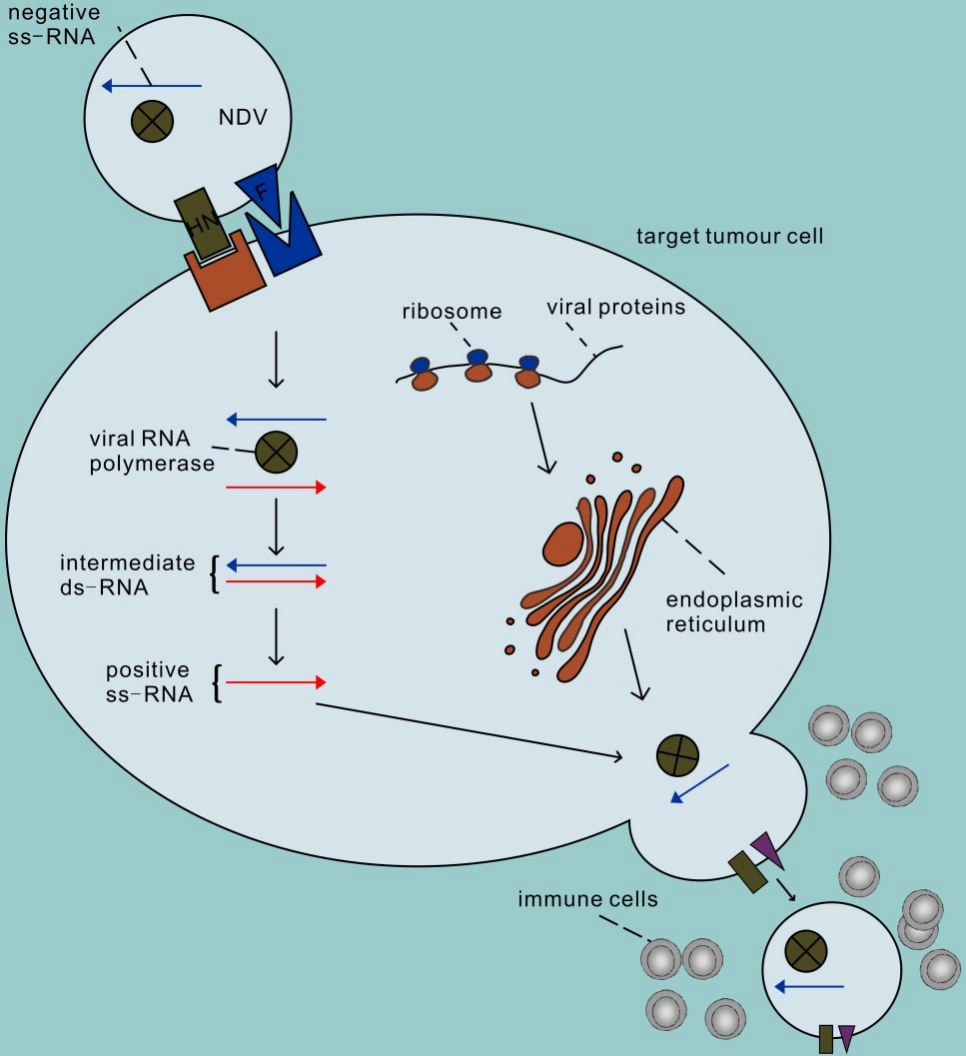
The process of infecting cells with NDV. In the first step, the NDV binds to sialic acid, a receptor on the tumour cell surface, via the HN protein, and then protein F initiates the fusion of the virus and host cell membranes. Secondly, the viral RNA polymerase transcribes the viral negative single-stranded RNA into positive single-stranded RNA as a template for mRNA and protein synthesis, and begins replication in the cytoplasm of the target cell. The surface proteins F and HN are processed in the rough endoplasmic reticulum, assembled on the host cell membrane after completion, and finally sprout to lead to new virions, initiating a new round of infection of tumour cells.

The second advantage of NDV is that it can efficiently and selectively replicate in tumour cells 17. The process of infecting cells with NDV is roughly divided into the following steps (Figure 1). First, the HN protein binds to sialic acid on the surface of host cells (such as the high-affinity receptor α 2,6-linked sialic acid) 18, and then the viral F protein is activated that mediates the fusion of virus and host cell membrane together with the HN protein. Therefore, the NDV genome enters the host cytoplasm to complete the transcription of the virus genome and the translation of virus proteins. In addition, NDV enters cells through caveolae-dependent or receptor-dependent endocytosis. According to recent research data 19, the endocytosis pathway may release the nucleocapsid by accelerating skeleton disintegration, and this effect will be enhanced at acidic pH. The second step of NDV infection involves virus replication. First the genes are transcribed in mRNA which are the templets for protein synthesis and then a full-length RNA molecule is synthesized (positive sense, antigenom) which is the template for genomic RNA molecules (negative sense). The offspring of the virus produced thereafter are released into the extracellular space with budding of the host cell, starting a new round of infection. Infections by NDV of cells induce a strong antiviral response. This antiviral response is initiated through the recognition of pathogen-associated molecules (PAMPs) by two types of pathogen recognition receptors: the Toll-like receptors (TLRs) such as TLR3 as well as the RIG-I-like receptors (RLRs) such as RIG-1 and MDA-5 20, 21. PAMPs are very common in pathogenic bacteria and viruses, including viral capsids, DNA, RNA, and viral protein products. For example, the RNA helicases RIGI and TLR3 recognize dsRNA, leading to the activation of interferon regulatory factor (IRF) 3, IRF 9 and nuclear factor kappa B (NF-κB) and subsequent production and release of type I/III IFN 22-24. IFN then activates host cells in an autocrine manner and activates surrounding cells in a paracrine manner to synthesize antiviral proteins, thus terminating the synthesis of cellular proteins and promoting rapid cell death and virus clearance. Therefore, normal cells are usually unable to enter the second step of virus replication. Unlike non-oncogenic cells, tumour cells show impaired antiviral interferon signalling, especially type I interferon signalling. This defect allows the virus to selectively infect tumour cells, which may be due to the dysregulation of interferon receptor expression, resulting in responsiveness to antiviral enzymes and damage to STAT1 signalling 25, 26. In addition, NDV has the ability to replicate in non-proliferative tumour cells, such as X-ray-irradiated tumour vaccine cells 27. Because viral replication in the cytoplasm is independent of cell proliferation, NDV can target tumour stem cells or tumour-dormant cells, both of which may be unaffected by chemotherapy or radiation therapy 28, 29. NDV can also replicate in apoptosis-resistant tumour cells 30, hypoxic cancer cells 31 and interferon-resistant tumour cells 32-35. Recent studies have shown that the V protein of NDV can target the reduction of STAT1 phosphorylation to antagonize the effect of interferon 36, which may be the reason why some tumour cells with antiviral INF signals can still be killed by NDV 37, 38.

In addition to its direct oncolytic effect, NDV can also promote the activation of the immune system to further antitumour activity 39-43. On one hand, NDV strongly stimulates tumour-associated immune cells, reversing the immunosuppressive state of the tumour microenvironment to an immune-activation environment. Significant increases in CD4+ and CD8+ T cell infiltration were observed in the tumour microenvironment 44, 45. More interestingly, upregulation of many immune checkpoint molecules was observed on both tumour-invasive T cell lines, including CTLA-4 and PD-1 46, 47. These findings suggest the possibility of combining NDV and immune checkpoint inhibitors to break immune resistance (discussed below). In fact, the increased antitumour efficacy of combination therapy with oncolytic virus and anti-CTLA-4, anti-PD-1, or anti-PD-L1 compared to treatment alone has been demonstrated in many studies 48-52. On the other hand, virus infection of tumour cells can induce the production of cytokines such as IFN-α, IFN-β, TNF-α and IL-1; thus, a large number of immune cells outside the tumour are induced to infiltrate into the tumour, where the activated nonspecific immune cells can kill and devour infected tumour cells that have not yet lysed or are resistant to viral oncolysis 53. Subbiah et al. injected NDV subcutaneously into tumour-bearing mice, and transcriptional profiles showed that genes related to the type I IFN response and a series of cytokines and chemokines were significantly upregulated, leading to the recruitment and proliferation of immune cells 35. As tumour cells are constantly attacked, they will gradually lyse and release a large number of tumour proteins. These tumour-specific antigens exposed to the tumour microenvironment can be recognized by antigen-presenting cells and induce T cells to attack tumour cells that are not infected with NDV. In bilateral flank syngeneic tumour models, the unilateral tumour injection of NDV LaSota strains found at the injection site and the distance of the injection site in the tumour microenvironment of CD4+ and CD8+ T lymphocytes were significantly increased 47. It is interesting to note that there is no this change observed in the spleen, which suggests that the activation of the T cell response is a tumour-specific, rather than because of nonspecific inflammation, suggesting the potential of NDV therapy to break immune resistance within the tumour. Although the inflammatory response caused by NDV infection helps the immune system clear the tumour, it also leads to the clearance of NDV by immune cells, thereby limiting the antitumour effect 25. Therefore, to optimize the antitumour effect, it is necessary to achieve a balance between sufficient viral replication, tumour lysis and immune response induction. According to the susceptibility of different tumour cells to NDV and differences in antiviral efficiency, the virus dose should be explored to gain more time for the virus to complete the mission of oncolysis.

## Engineered NDV as an improved cancer vaccine

With the emergence of cell culture techniques in 1940 54 and molecular cloning and genetic engineering in 1970 55, it became possible to manipulate genes at the molecular level, and genetically modified Newcastle disease virus came into being, bringing the application of NDV to a new stage. The genomic RNA of NDV and the NP, P, and L proteins together constitute the nuclear protein complex. With the initiation of the first round of RNA transcription and the translation and synthesis of viral proteins, infectious progeny viruses are produced. According to this principle, in 1999, European scholars established the Newcastle disease virus reverse genetic operating system (RGS) for the first time 33, 56. The modification of virus genes through the RGS system, such as eliminating some nonessential genes of the virus or inserting exogenous functional genes, can reduce the pathogenicity of the virus, improve the specificity of the virus for tumour infection and enhance the activation ability of the immune system. In 2000, researchers first tried to construct recombinant NDVs expressing foreign genes 57. The results showed that recombinant NDV could stably express foreign proteins, but it led to growth retardation of the recombinant virus. This may be related to the fact that the inserted gene fragment was too large to exceed the carrying capacity of the viral vector genome and the insertion between NDV HN and L genes is not an optimal insertion site. Nevertheless, their research still indicated the potential of NDV as a vaccine vector 57.

With further research, researchers found that all of the NDV genomes conform to the “rule of six” specific to APMV-1, with a capacity of more than 5 kb to integrate transgenes 58, 59, and the insertion site of foreign genes in the NDV genome is a key issue for optimizing gene expression and immunogenicity. To determine the optimal insertion of exogenous genes, some researchers inserted EGFP and IL2 into five different gene spacer regions of the NDV genome: the NP and P genes, the P and M genes, the M and F genes, the F and HN genes, and the HN and L genes. They found that the mRNA and protein expression levels were the highest when the target gene was inserted between NP/P sites 60, which may be related to the nonequal and progressive decline of transcription of the viral genomic RNA from 3' to 5'. However, according to the research results of Zhao et al. 61, it is better to insert GFP between P/M sites than between NP/P sites. This disproportionate effect is probably because GFP gene insertion changes the ratio between the NP and P proteins. In vesicular stomatitis virus, it has been proven that maintaining the specific ratio of NP and P proteins is the best method to support efficient replication and encapsulation 62, 63. Thus, the P/M site is recommended as an ideal insertion site.

At present, in most vaccines based on NDV vectors, inserting foreign genes into the P/M site as an independent transcription unit (ITU) is the most commonly used modification method, but this method will weaken the transcription of downstream genes and viral replication. To overcome this shortcoming, Zhang et al. 64 proposed the strategy of using an internal ribosomal entry site (IRES) to bring foreign genes into the optimal insertion site of NDV, making foreign genes the second open reading frame (ORF) of the virus genome. IRES can promote the internal initiation of RNA translation to promote the expression of two or more proteins of polycistronic transcription units in eukaryotic cells. Therefore, this strategy ensures the expression of foreign proteins and the replication of viruses. He et al. 65 also confirmed the above view by combining IRES and ITU to recombine NDV to express REF and EGFP in 2020, suggesting that the combination of two or more foreign proteins expressed in this way is a promising and feasible approach.

To further optimize the oncolytic effect of NDV, we can enhance the body's antitumour immune response by engineering NDV to express specific promoters, cytokines, antibodies or tumour antigens. Some of these strategies are reviewed below. It is well known that excessive expression of tumour-associated antigens (TAAs) is an important indicator to differentiate from normal cells in the process of tumorigenesis and development. Therefore, engineering NDV to express tumour-specific and/or tissue-specific gene promoters, such as high molecular weight mucin-like glycoprotein, DF3 (MUC1), prostate-specific antigen (PSA) and alpha fetoprotein (AFP), can optimize the targeting effect of NDV and limit virus replication to specific tumour cells. Chen L et al. 66 constructed a recombinant adenovirus containing the DF3/MUC1 promoter, which showed high selectivity in a breast cancer model. And the antitumour activity of the vector was enhanced by limiting the expression of “suicide genes” to DF3 overexpressed breast cancer cells, such as herpes simplex thymidine kinase (HSV-tk) which can activate a prodrug within tumor cells and thereby render the tumour cells sensitive to agents. Recently, researchers have found that human telomerase reverse transcriptase promoter (hTERT) is more commonly expressed in more types of tumours. hTERT is necessary for telomere extension and usually exists at a high level only in rapidly dividing cells. Using hTERT as a promoter to increase the targeting of NDV has been proven in recombinant adenoviruses expressing HN of NDV 67. In addition, through genetically engineered mutation and/or deletion of some genes that play an important role in the replication of the virus in normal cells but are insignificant for the replication of the virus in tumour cells, specific attenuated strains can also be obtained so that the expression of key genes of the virus is limited to within tumour cells. Subbiah et al. 35 obtained a type-1 interferon sensitive recombinant NDV by abrogating its V protein expression, resulting in virus replication in tumor cells with IFN defects but with reduced or crippled virus replication in normal cells.

T cells are important effector cells of antitumour immunity, so enhancing the T cell response is key to the treatment of tumours 68-70. The expression of cytokines, related ligands or checkpoint blockers by engineered NDV can effectively increase local immune cell infiltration and enhance the body's antitumour immunity (Table 1). Cytokines are small molecular proteins with extensive biological activity that are synthesized and secreted by immune cells after stimulation, and they can directly stimulate immune effector cells in the tumour microenvironment or recruit more immune cells into the tumour area to improve the antitumour response of cytotoxic effector cells 71. So far, many studies have constructed NDV to express interferon and pro-inflammatory cytokines, such as rNDV-INF 72, rNDV-IL24 73, rNDV-IL2 74, 75 and rNDV-IL12 76. These recombinant NDVs showed stronger antitumour effects than those of wild type NDVs *in vitro* and *in vivo* in animal models. This way of expressing cytokines that can promote the T cell response through recombinant NDV is similar to creating an “armed” oncolytic virus, which can induce tumour cell apoptosis and enhance T cell activation and function.

However, in the tumour microenvironment, activated T cells enhance the immune response and regulatory T cells (Tregs). Tregs can inhibit the effector T cell response by secreting inhibitory cytokines, so they are easily used by tumour cells and lead to immune escape, but checkpoint inhibitors can effectively block this situation 77. At present, immune checkpoint blockers such as ipilimumab and nivolumab have been approved for the clinical treatment of tumours. However, some patients did not show the expected effect after treatment, mainly due to the lack of target protein expression or the lack of infiltrating T cells in the tumour. From the perspective of therapeutic principles, the combination of oncolytic viruses and immune checkpoint blockers has good complementarity. On one hand, NDV induces the upregulation of immune checkpoint molecules, and on the other hand, NDV induces an inflammatory response, leading to the infiltration of a large number of immune cells. Zamarin et al. 47 injected NDV before using a CTLA-4 inhibitor in a mouse melanoma model. NDV infection not only induced a local inflammatory response in tumors but also increased the inflammatory response and antitumor response of distant uninfected tumor sites, which effectively enhanced the efficacy of checkpoint inhibitors. Similar results have been reported in studies using the combination of PD-1/PDL1 blockade and NDV 46. Although the combination of NDV and immune checkpoint blockers can solve the problem of local immune resistance, the problem of serious immune-related adverse events (irAEs) may still exist when using checkpoint blockers. irAEs have been reported in up to 10% of patients receiving CTLA-4 or PD-1/PD-L1 blockers. Based on the aforementioned experimental data, Gayathri et al. 78 constructed recombinant viruses expressing checkpoint inhibitor antibodies (rNDV-anti-PD1 and rNDV-anti-PDL1) with NDV as the vector for the first time and tested the therapeutic effect on a mouse melanoma model by administering the virus with different combinations of systemic CTLA4 blockers. Compared with a single agent, the combination therapy enhances the antitumor effect and results in a longer survival time. Thus, the delivery mode of the NDV vector limits the effect of the checkpoint blocker to the local tumor and reduces systemic side effects, which further expands the method of NDV recombination and is a developmental direction that should be explored in the future.

## Clinical experience with NDV

As a tumour vaccine, NDV has gone through the following stages. In 1965, Cassel and his colleagues 2 first injected the 73T strain of NDV directly into the tumour area of patients with cervical cancer. The results showed extensive tumour shedding and a reduction in supraclavicular lymph node metastasis, which preliminarily proved the antitumour potential and safety of NDV. Subsequently, Cassel proposed the strategy of using autologous or allogeneic viral oncolysate (VOL) as a vaccination. In the postoperative treatment of stage II malignant melanoma 79, viral oncolysate of NDV was used as an adjuvant immunotherapy. In the 10-year observation of 83 patients, more than 60% of the patients survived without recurrence. In 1979, Kobayashi from Japan proposed the concept of “viral xenogenization” and confirmed that the immunogenicity of live cell vaccines is higher than that of tumour lysates 80. From 1990 to 2008, the team led by Schirrmacher was working to develop a safe and effective autologous tumor vaccine (ATV) 27, which was composed of 10 million irradiated tumour cells that were pre-infected with the attenuated NDV strain Ulster. They used the vaccine in eight different types of cancer: breast cancer, colon cancer, rectal cancer, kidney cancer, head and neck cancer, pancreatic cancer and glioblastoma multiforme. The results showed that the use of a high-quality ATV-NDV vaccine can obviously improve the survival prognosis of patients. The median progression-free survival (PFS) and median overall survival (OS) were two times higher in the control group, and no serious side effects were observed. The biopsy of the tumour response of memory T cells showed significant increases, indicating that after inoculation, cell culture with ATV NDV is feasible and safe 27.

The support of early clinical research data has laid a foundation for the further development of NDV treatment strategies. How to combine NDV with other antitumour treatment methods and fully mobilize the body's antitumour response is an important topic in the field of NDV antitumour research at present and in the future. In 2015, to further improve the efficacy of the ATV vaccine, Schirrmacher et al. 81 added an NDV-specific single chain antibody (bsHN-CD28) with dual specificity to the surface of ATV-NDV. The construct bsHN‑CD28 bind with one arm to the HN molecule of the NDV and the second arm is directed against CD28, an important molecule on t cells to deliver costimulatory signals. After 14 patients with advanced colorectal cancer were treated with the ATV-NDV vaccine supplemented with bsHN-CD28, no serious adverse events occurred. At least one immune response of tumour-reactive T cells was observed in all patients during the five vaccinations. This study shows that the three-component vaccine is safe and that adding a T cell costimulatory signal to the surface of ATV-NDV can reactivate T cells that may have been activated in advanced cancer.

Another new strategy was developed by researchers from the Immunological and Oncological Center (IOZK) in Cologne, Germany. These researchers proposed that the combination of viral oncolysates (VOL) with DC cells (DCs) can enhance the antigen presentation ability of DCs, thus inducing T cell proliferation and cytokine secretion. In a case report 82, a patient with hormone refractory metastatic prostate cancer was treated with local hyperthermia (LHT) combined with VOL-DCs to achieve complete remission after the failure of standard treatment, which confirmed that hyperthermia and Vol pre-treatment can activate the immune system and induce VOL reactive CD4+ T helper cells. After DC vaccination for more than three years, the patient was found to have a good antitumour memory T cell response. In another case report 83, a 70-year-old patient with invasive ductal breast cancer and primary liver metastasis survived for more than 66 months after LHT, Vol and DC treatment, and no recurrence or further metastasis occurred during the treatment. These cases show that this combination therapy is not limited to specific tumour types, and further exploration of this therapy is necessary.

Immune checkpoint inhibitors have been among the most promising research directions in the field of tumour therapy in recent years. However, clinical results show that immune checkpoint blockers have a good effect on only some patients with solid tumours, but the effect on most patients with solid tumours is not obvious. To compensate for this limitation, researchers began to try to combine oncolytic viruses and immune checkpoint inhibitors. At present, the clinical evaluation of recombinant NDV (73-T strain) expressing granulocyte-macrophage colony-stimulating factor (GM-CSF) combined with durvalumab for the treatment of various advanced malignant tumours is underway (NCT03889275). The results show that the secretion of pro-inflammatory cytokines, such as IL-6, IL-8 and IFN-α, in human PBMCs is significantly increased, which stimulates the maturation of DCs and enhances the antitumour response 84. In addition, the combination therapy of other oncolytic viruses and immune checkpoint inhibitors has also achieved good clinical results, such as HF10, which is a spontaneously mutated oncolytic virus derived from a herpes simplex virus-1 (HSV-1), combined with nivolumab in the adjuvant treatment of melanoma (NCT03259425) and talimogene laherperepvec (T-VEC) combined with pembrolizumab in the treatment of advanced malignant melanoma (NCT02263508). These clinical data illustrate the potential of NDV infection to break the tolerance of immune checkpoint inhibitors.

## Summary

NDV has been used for more than 60 years as an oncolytic vaccine to improve antitumour efficacy. In the past 60 years, the development of NDV has experienced many obstacles and made many breakthroughs. Preclinical and clinical experimental data show that NDV can not only cause cancer cells lysis but can also induce long-lasting antitumour immunity. Recently, it has been found that NDV can effectively compensate for the limitations of immune checkpoint blockers. This review mainly summarizes the positive clinical results of each developmental stage to guide further research and allow more cancer patients to benefit from NDV earlier.

Although NDV has great advantages and potential in improving cancer treatment, many questions remain unanswered, including the ideal strategy of genetic engineering and the best combination of immune checkpoint inhibitors. Although the current research data provide some references for these problems, most studies have not explored the heterogeneity of human cancer. Therefore, in the context of such clinical trials, we must collect as much information as possible, understand the evolution of the immune responses to viruses and tumours, and guide more research to promote the further development of NDV as an individualized oncolytic vaccine.

## Figures and Tables

**Table 1:** Preclinical studies of recombinant Newcastle disease virus (rNDV) (2016-2020)

Type of rNDV	Tumour type	Immune responses	Reference
rNDV-anti-CD28-IL12+anti-CTLA4	Melanoma	↑IFN-α, granzyme B; ↑TILs (both in treated and nontreated tumors)	78
rNDV-anti-PDL1-IL12+anti-CTLA4	Melanoma	↑IFN-α; ↑TILs (both in treated and nontreated tumors)	
rNDV-IL12-IL2	Hepatoma	↑IFN-γ, IP-10	75
rNDV-IL12	Colon cancer	↑BAX, p53; ↑IL-2, IL-12, IFN-γ; ↓KRAS, BRAF; ↓MAPK1, Notch1, MCP-1, VEGF	85
rNDV-IL12	Colon cancer	↑IL-2, IL-12, IFN-γ; ↑Fas, BAX, Smad3, BID, granzyme B, caspase 8	86
rNDV-IL2	Hepatoma	↑IL-2; ↑TILs	74
rNDV-IL15	Melanoma	↑IL-15; ↑NK cells, TILs	87
rNDV-IL15-IL7	Melanoma	↑IL-15, IL-7; ↑NK cells, TILs	88
rNDV-IFN-λ1	Lung cancer	↑IFN-λ1; ↑GRP78, CHOP, p-eIF2α, beclin1, LC3, caspase 3; ↑NK cells	89
rNDV-TRAIL	Hepatoma	↑TRAIL; ↑caspase 3; ↑TILs	90
rNDV-RVG	Gastric cancer	↑E-cadherin, RVG; ↓N-cadherin, vimentin, α7-nAChR, P-MEK, P-ERK	91
rNDV-P53	Hepatoma	Reduce the mitochondrial membrane potential	92

Abbreviations: IL: interleukin; CTLA4: cytotoxic T cell related protein-4; ↑: upregulated; ↓: downregulated; IFN: interferon; TILs: tumour infiltrating lymphocytes; PDL1: programmed cell death ligand 1; IP10: interferon-γ inducible protein 10; MAPK1: mitogen-activated protein kinase 1; Notch1: notch homolog 1; MCP-1: monocyte chemoattractant protein-1; VEGF: vascular endothelial growth factor; NK: natural killer cell; GRP78: glucose-regulated protein 78; CHOP: C/EBP-homologous protein; eIF: eukaryotic translation initiation factor; LC3: Microtubule associated protein I light chain 3; TRAIL: TNF-related apoptosis-inducing ligand; RVG: rabies virus glycoprotein; nAChR: nicotinic acetylcholine receptor; P-MEK/P-ERK: phosphorylated MEK/ERK protein.
